# Characterization of the Tissue and Strain-Specific Microbiota of *Anopheles funestus* Giles (Diptera: Culicidae)

**DOI:** 10.3390/tropicalmed9040084

**Published:** 2024-04-13

**Authors:** Chia-Yu Chen, Wai-Yin Chan, Arshad Ismail, Shüné V. Oliver

**Affiliations:** 1 Wits Research Institute for Malaria, School of Pathology, Faculty of Health Sciences, University of the Witwatersrand, Johannesburg 2193, South Africa; shuneo@nicd.ac.za; 2 Centre for Emerging Zoonotic and Parasitic Diseases, National Institute for Communicable Diseases, Division of the National Health Laboratory Service, Johannesburg 2193, South Africa; 3 Sequencing Core Facility, National Institute for Communicable Diseases, Division of the National Health Laboratory Service, Johannesburg 2193, South Africa; chan.wya@gmail.com (W.-Y.C.); arshadi@nicd.ac.za (A.I.); 4 Department of Biochemistry, Genetics and Microbiology (BGM), Forestry and Agricultural Biotechnology Institute (FABI), University of Pretoria, Pretoria 0028, South Africa; 5 Department of Biochemistry and Microbiology, Faculty of Science, Engineering and Agriculture, University of Venda, Thohoyandou 0950, South Africa; 6 Institute for Water and Wastewater Technology, Durban University of Technology, Durban 4000, South Africa

**Keywords:** mosquito, vector control, microbiota, midgut, ovaries, salivary glands, paratransgenesis, 16S rRNA

## Abstract

The mosquito microbiota is a critical determinant of mosquito life history. It is therefore a target for novel vector control strategies like paratransgenesis. However, the microbiota in *Anopheles funestus*, a major African malaria vector, is poorly characterized. Thus, the study aimed to investigate the overall bacterial landscape in the salivary glands, ovaries and midguts of three laboratory strains of *An. funestus* differing in insecticide-resistant phenotype by sequencing the V3–V4 hypervariable region of bacterial 16S rRNA genes. When examining alpha diversity, the salivary glands harbored significantly more bacteria in terms of species richness and evenness compared to ovaries and midguts. On the strain level, the insecticide-susceptible FANG strain had significantly lower bacterial diversity than the insecticide-resistant FUMOZ and FUMOZ-R strains. When looking at beta diversity, the compositions of microbiota between the three tissues as well as between the strains were statistically different. While there were common bacteria across all three tissues and strains of interest, each tissue and strain did exhibit differentially abundant bacterial genera. However, overall, the top five most abundant genera across all tissues and strains were *Elizabethkingia*, *Acinetobacter*, *Aeromonas*, *Cedecea* and *Yersinia*. The presence of shared microbiota suggests a core microbiota that could be exploited for paratransgenesis efforts.

## 1. Introduction

The mosquito genus *Anopheles* includes 465 formally recognized species, of which 70 species can transmit human malaria parasites and 41 species are considered to be dominant vector species [1,2]. *Anopheles gambiae s.s*, *Anopheles arabiensis* and *Anopheles funestus* are major malaria vectors in Africa [1,2]. *An. funestus* in particular is an extremely efficient vector of malaria, often exhibiting high entomological inoculation rates. For example, in Northwest Tanzania, *An. funestus s.l*. represented 94.5% of all vectors and was responsible for 96.5% of malaria transmission, with a sporozoite rate of 3.4% and average monthly entomological inoculation rate of 4.57 per house [3]. Furthermore, 92.9% of the *An. funestus s.I.* sampled in Northwest Tanzania was predominated by *An. funestus s.s*. [3]. This is because *An. funestus* is highly endophagic and endophilic, with strong anthropophilic tendencies, and it is believed to be the first species of *Anopheles* to specialize in biting humans [4,5].

Although the endophilic and endophagic habits of *An. funestus* make it suitable for traditional vector control methods such as indoor residual spraying and insecticide-treated nets, this is hampered by large-scale pyrethroid resistance. Pyrethroid resistance contributed to a major malaria epidemic in South Africa in 2000 [6]. Since then, reports of metabolic pyrethroid resistance have continued throughout Africa [7,8,9].

Due to insecticide resistance in *An. funestus*, it is becoming critical to seek out novel, non-insecticidal interventions to deal with this vector. A strategy for eliminating malaria transmission or reducing vector competence involves the genetic modification of malaria vectors. Alternatively, rather than genetically modifying the mosquito, bacterial symbionts found within the mosquito core microbiota can be genetically engineered to produce anti-*Plasmodium* molecules. The use of these bacterial symbionts, whether genetically engineered or native to the mosquito, to inhibit *Plasmodium* transmission is known as paratransgenesis [10,11].

Key to paratransgenesis and genetic interventions is the understanding of the immune response of mosquitoes, because the immune system of mosquitoes affects vector competence in *Anopheles* mosquitoes [12,13]. Most importantly, bacteria within the core midgut microbiota of mosquitoes can modulate the responses of mosquitoes to *Plasmodium* by blocking *Plasmodium* infection.

The core midgut microbiota of *Anopheles* mosquitoes has been reported to be dominated by Gammaproteobacteria (*Acinetobacter*, *Aeromonas*, *Pantoea*, *Pseudomonas*, *Enterobacter* and *Serratia),* Flavobacteriia (*Elizabethkingia*) and Alphaproteobacteria (*Asaia*) [10,13,14,15]. Thus, interest has been growing, and researchers have sought to establish efficient paratransgenesis tools from these core bacteria. For example, *Asaia* has been reported to be part of the core microbiota in *Aedes* sp., *Anopheles* sp., and *Culex* sp. in several tissues (midgut, testes, ovaries and salivary glands) [16,17,18,19,20,21]. Moreover, interestingly, *Asaia* has been reported to prime the immune responses of *Anopheles stephensi* and *Anopheles gambiae*, thereby preventing the development of the *Plasmodium* parasite [22,23]. *Enterobacter* isolated from the midguts of wild *Anopheles arabiensis* from Zambia has been shown to enable 99% resistance to *Plasmodium falciparum* in *An. stephensi* and *An. gambiae* by synthesizing reactive oxygen species, which inhibits *Plasmodium* oocyst formation [24]. Similarly, recombinant *Pantoea agglomerans* (previously known as *Enterobacter agglomerans*), modified from the naturally occurring *P. agglomerans* mosquito midgut symbiont to express antimalarial effector molecules, successfully suppressed *P. falciparum* and *Plasmodium berghei* in both *An. gambiae* and *An. stephensi* [25].Furthermore, *Serratia* strains isolated from *An. stephensi* ovaries have been genetically engineered to express multiple antimalarial effector molecules, and have been successful in inhibiting *Plasmodium* oocyst loads by 92 to 93% in *An. gambiae* [26].

With the problem of insecticide resistance being apparent, it is crucial to consider the effect of insecticide resistance on the composition of the microbiota. Previous studies have demonstrated changes in the gut microbiota associated with insecticide-resistant anophelines [27,28,29,30]. Therefore, in this study, the microbiota of three laboratory strains of *An. funestus* differing in insecticide-resistant phenotype and intensity was characterized.

In the context of paratransgenesis and vector competence, the elucidation of the role of microbial communities in the salivary glands of mosquitoes is equally as important as that of the midgut, since salivary glands, together with the midgut of mosquitoes, are essential for the digestive system of the mosquito [31]. Furthermore, a successful paratransgenic tool would require the ability to be passed on both vertically and horizontally within a mosquito colony, so that the anti-*Plasmodium* properties could persist within a population [11]. Therefore, the evaluation of the microbiota of ovaries is also important. Thus, in addition to evaluating the microbiota of different *An. funestsus* strains, the microbiota from midguts, salivary glands and ovaries was also compared in order to establish a core microbiota of *An. funestus* that may offer potential candidates for paratransgenesis.

## 2. Materials and Methods

### 2.1. Mosquitoes

Three laboratory strains of *An. funestus* were used in this study: FUMOZ, FUMOZ-R and FANG. The FUMOZ strain originated from Southern Mozambique in 2000 [32]. The FUMOZ-R strain was selected from FUMOZ by λ-cyhalothrin exposure [32]. Both FUMOZ and FUMOZ-R display pyrethroid resistance as well as carbamate resistance [33]. The FANG strain was colonized from Calueque, southern Angola in 2002 and is fully susceptible to all insecticides [34]. All strains were housed in the Botha de Meillon insectary in Sandringham, Johannesburg, and were reared according to the methods described by Hunt et al. [34]. In brief, this involves a 12:12 h light cycle with a 30 min dusk/dawn cycle with temperatures at 25 ± 2 °C and 80 ± 5% humidity. Adults that were three days old and had not been blood-fed were used for midgut dissections. The salivary glands and ovaries were dissected from female adults that were 15 days old and had had 3 blood meals. Blood was provided by a consenting volunteer (author S.V.O) on days 3, 7 and 11 as per the animal ethics waiver (number: S Oliver 03-01-2018). Female adults were allowed to feed until repletion. Only females that were fully fed after each blood meal were allowed to continue to the next blood meal, and only females fed fully for all three blood meals were considered for analysis. Dissections were performed on day 15 to ensure that the blood was fully digested and that no blood remained in the gut prior to dissections.

### 2.2. Tissue Dissections and DNA Extraction

Following the ethanol-killing of mosquito specimens, midguts, ovaries and salivary glands were dissected from each *An. funestus* strain following aseptic procedures. Dissections of midguts were performed in replicates of five with five midguts pooled as one replicate making the total number of mosquitoes 25. The same was done for the dissections of ovaries. DNA extraction of the pooled tissues was also performed in replicates of five, making it a total number of 25 mosquitoes for midgut and 25 mosquitoes for ovaries per mosquito strain. Salivary glands were pooled as 10 tissues per tube for three replicates of DNA extraction, making the total number 30 salivary glands per mosquito colony. The dissected tissues were pooled directly into buffer ATL and proteinase K, provided by the DNeasy Blood & Tissue Kit (Qiagen, Hilden, North Rhine-Westphalia, Germany). Total genomic DNA was extracted using the DNeasy^®^ Blood & Tissue kit (Qiagen: 69506, Germany). Total genomic DNA extraction was carried out following an overnight incubation, as recommended in the user’s manual. The overnight incubation ensured the tissues were fully homogenized. A buffer-only negative control was subjected to the same DNA extraction procedure.

### 2.3. DNA Amplification and Sequencing

Total genomic DNA of the midguts, ovaries and salivary glands of all three *An. funestus* strains were PCR-amplified using universal bacterial primers targeting the V3–V4 hypervariable region of the bacterial 16S rRNA gene [35]. The primer sequences were as follows: forward primer 5′ GTC TCG TGG GCT CGG AGA TGT GTA TAA GAG ACA GGA CTA CHV GGG TAT CTA ATC C 3′ and reverse primer 5′ TCG TCG GCA GCG TCA GAT GTG TAT AAG AGA CAG CCT ACG GGN GGC WGC AG 3′ (4 nmol Ultramer^®^ DNA Oligo 55 bases: Integrated DNA Technologies, Coralville, IA, USA). A positive control containing known bacterial species (ZymoBIOMICS^®^ Microbial Community Standard, D6300, Irvine, CA, USA) and two negative controls (a non-template and an extraction buffer-only) were also amplified by PCR. Library preparation was performed according to the standard instructions of the 16S Metagenomic Sequencing Library Preparation protocol (Illumina TM, San Diego, CA, USA). Libraries were then sequenced on the MiSeq platform using MiSeq Reagent kit v3 (Illumina, USA). Paired-end 2 × 300 bp sequencing was performed at the Sequencing Core Facility, National Institute for Communicable Diseases. The buffer-only negative control was also amplified and sequenced.

### 2.4. Bioinformatics

Sequences were first filtered and pair-end trimmed using trimGalore (v0.6.5-1) to remove the Nextera adapter sequences. Quality control was then performed using MultiQC (v1.6) to ensure clean data were used for downstream analysis. All the downstream analyses were performed in R studio (v4.2.1), including classification, abundance estimations, statistical analysis, and visualization. Clean reads were pre-processed using the DADA2 package (v1.24.0), including quality inspection, filtering, trimming, dereplication, sample inference, merging paired-end reads and removal of chimeric sequences [36].

Taxonomy was assigned to the obtained amplicon sequence variants (ASVs) and the ASV abundance estimates were determined using training sequence sets based on the SILVA reference database (v138; https://zenodo.org/record/4587955#.Y9JGXnZBxPY, accessed 29 November 2023). DADA2 outputs were then constructed into phyloseq objects using the phyloseq package (v1.40.0) [37]. The phyloseq objects were then used for further analyses. For alpha diversity, species richness was determined using the Chao1 index, while relative abundance was measured using the Shannon index. The Wilcoxon rank-sum tests were used to compare alpha diversity between the tissues. For beta diversity, ordination plots were constructed using the Nonmetric Multidimensional Scaling (NMDS) method. The data clustering between the three different tissues for the NMDS plot was statistically assessed using a PERMANOVA (permutation test with pseudo-F ratios) as implemented by the adonis function in the vegan package (v2.6-4; https://github.com/vegandevs/vegan, accessed 8 February 2024). Barplots showing the top 5 most abundant genera ranked across the different tissues were also constructed. To visualize shared microbial communities between the different genera and species, UpSet plots were generated using Venn Diagram (v1.7.3) and UpsetR (v1.4.0) [38]. Differential abundance analysis between sample groups was performed using DESeq2 (v1.24.0) [39]. Except for the UpSet plots, all plots were constructed using ggplot2 (v3.4.0) [40].

## 3. Results

### 3.1. Alpha Diversity

**Figure 1:**
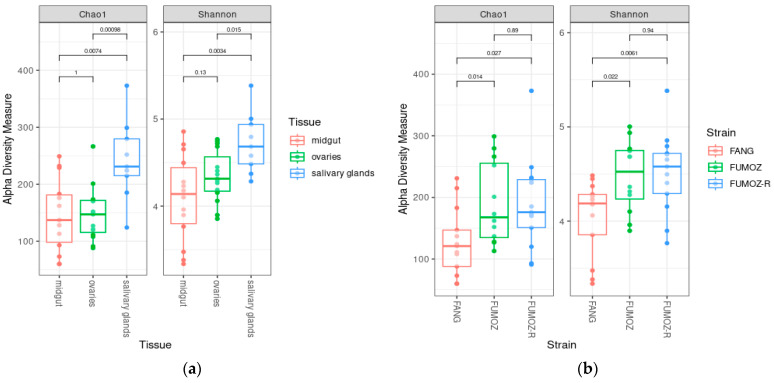
Alpha diversity of the tissue- and strain-specific microbiota of *An. funestus*: (**a**) alpha diversity of three different tissues (midgut, ovaries and salivary glands) of *An. funestus*; (**b**) alpha diversity of three different *An. funestus* strains (FANG, FUMOZ and FUMOZ-R) across all three tissues. The *p*-values were determined by the Wilcoxon rank-sum test.

The Chao1 index measures species richness (total number of species in the different tissues). The highest numbers of species were found in the salivary glands according to the Chao1 index (Figure 1a). Furthermore, the number of species found in the salivary glands was significantly higher than that found in the ovaries and midguts, since the Chao1 indices of species richness indicated a significant difference between salivary glands and the midguts (Wilcoxon rank-sum test, *p* = 0.007) as well as between the salivary glands and the ovaries (Wilcoxon rank-sum test, *p* = 0.001) (Figure 1a). By contrast, no significant difference was indicated by the Chao1 index between the midgut and ovaries (Wilcoxon rank-sum test, *p* = 0.100) (Figure 1a). Similar findings were observed for species evenness (relative abundance of species in the different tissues), which is measured by the Shannon index. The Shannon index in the salivary glands was the highest compared to that of the ovaries and midguts, and was significantly different from the species evenness found in the ovaries and midguts, since the Shannon index of species evenness indicated a significant difference between the salivary glands and the midguts (Wilcoxon rank-sum test, *p* = 0.003) as well as between the salivary glands and the ovaries (Wilcoxon rank-sum test, *p* = 0.015) (Figure 1a). By contrast, no statistical difference in the Shannon index was observed between the midguts and ovaries (Wilcoxon rank-sum test, *p* = 0.130) (Figure 1a). In terms of the alpha diversity between the three An. funestus strains, statistical differences were found between FANG and FUMOZ when looking at the Chao1 index (Wilcoxon rank-sum test, *p* = 0.014) and the Shannon index (Wilcoxon rank-sum test, *p* = 0.022, Shannon). Similarly, statistical differences were found between FANG and FUMOZ-R when looking at the Chao1 index (Wilcoxon rank-sum test, *p* = 0.027) and the Shannon index (Wilcoxon rank-sum test, *p* = 0.006, Shannon) (Figure 1b). However, no statistical difference was found between FUMOZ and FUMOZ-R for both the Chao 1 index (Wilcoxon rank-sum test, *p* = 0.890) and the Shannon index (Wilcoxon rank-sum test, *p* = 0.940) (Figure 1b). Furthermore, both Chao1 and Shannon indices for FANG were the lowest compared to FUMOZ and FUMOZ-R.

### 3.2. Beta Diversity

**Figure 2:**
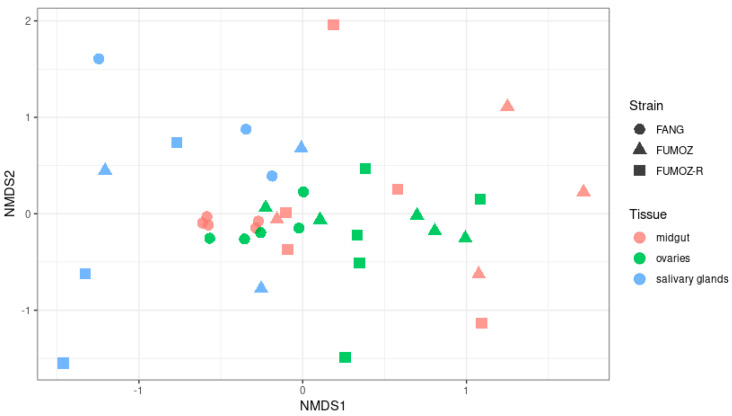
Beta diversity of the microbiota in the midgut, ovaries and salivary glands of *An. funestus* plotted as an ordination plot using the Nonmetric Multidimensional Scaling (NMDS) method based on the Bray − Curtis distance measurement. The stress value was 0.23. The PERMANOVA was determined using the adonis function in the vegan package, where *p* = 0.001 for tissues, *p* = 0.001 for strain and *p* = 0.006 for tissue:strain.

Beta diversity measures the diversity between samples, and has been visualized as a Nonmetric Multidimensional Scaling (NMDS) ordination plot. The NMDS plot shows a slight overlapping of diversity between the three *An. funestus* tissues of interest (midgut, ovaries and salivary glands) and between strains (FANG, FUMOZ and FUMOZ-R). However, overall, there was a distinct and statistically significant clustering of diversity between the different tissues (PERMANOVA, *p* = 0.001) and strains (PERMANOVA, *p* = 0.001) (Figure 2). There was also a statistically significant interaction between the tissues and strains (PERMANOVA, *p* = 0.006).

### 3.3. Shared Bacteria between the Different Tissues and Strains of An. funestus

**Figure 3:**
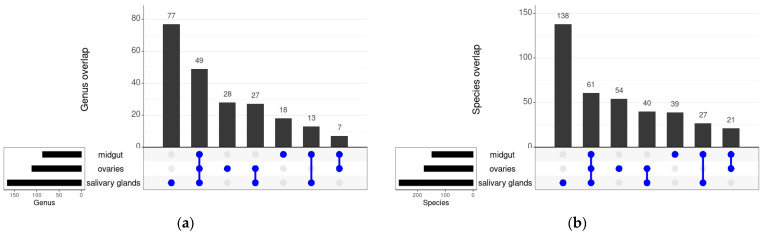

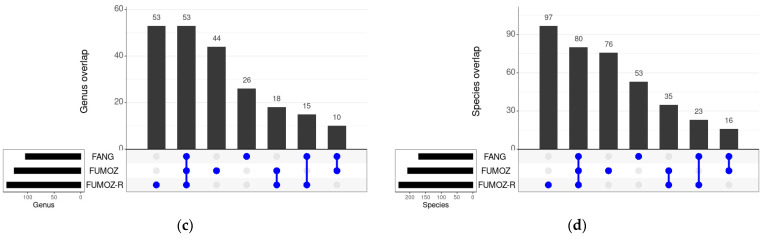
Shared bacteria between the different tissues and strains of *An. funestus:* (**a**) shared genera between three tissues; (**b**) shared species between three tissues; (**c**) shared genera between three strains; (**d**) shared species between three strains. Individual dots indicate numbers of individual genera or species. Joined dots indicate numbers of shared genera or species between the tissues or strains.

A total of 49 bacterial genera were shared between all the tissues across the three *An. funestus* strains (FANG, FUMOZ and FUMOZ-R), which encompassed 61 species (Figure 3a,b). Ovaries and salivary glands shared the most bacterial genera (27) and species (40), while midguts and ovaries shared the least bacterial genera (7) and species (21) (Figure 3a,b). In terms of *An. funestus* strains, 53 bacterial genera and 80 bacterial species were shared across all three strains (FANG, FUMOZ and FUMOZ-R). FUMOZ and FUMOZ-R shared the most bacterial genera (18) and species (35), while FANG and FUMOZ shared the least bacterial genera (10) and species (21) (Figure 3c,d). The full list of shared bacterial genera and species can be found in Supplementary Tables S1–S4.

### 3.4. Relative Abundance

**Figure 4:**
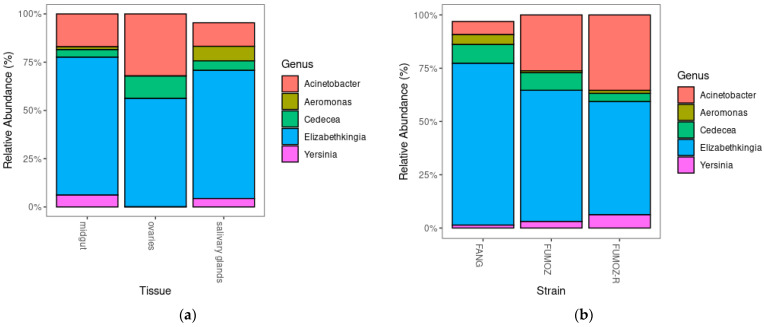
Relative abundance of the top 5 ranked genera in *An. funestus*: (**a**) relative abundance of bacterial genera in the midguts, ovaries and salivary glands; (**b**) relative abundance of bacterial genera in FANG, FUMOZ and FUMOZ-R.

The overall top 5 most abundant genera in terms of percentages of total microbiota across the three tissues of interest and all three strains of *An. funestus* were *Elizabethkingia*, *Acinetobacter*, *Aeromonas*, *Cedecea* and *Yersinia* (Figure 4). The full list of micriobiota found across the three *An. funestus* tissues and strains as well as the top 50 most abundant taxa can be found in Supplementary Tables S5 and S6, respectively.

### 3.5. Differential Abundance

**Figure 5:**
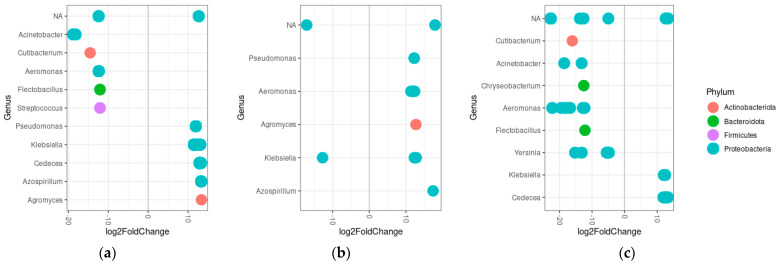
Differential abundance of genera in *An. funestus* tissues. (**a**) Differential abundance between midgut (left) and salivary glands (right). (**b**) Differential abundance between midgut (left) and ovaries (right). (**c**) Differential abundance between ovaries (left) and salivary glands (right). Numbers on either side of the 0 represent log2fold change of bacterial genera that are significantly different at an alpha value of 0.01. Each dot represents a single OTU.

The midguts of *An. funestus* appeared to be more dominated by *Acinetobacter*, *Cutibacterium*, *Aeromonas*, *Flectobacillus* and *Streptococcus* compared to the salivary glands, while the salivary glands of *An. funestus* were mostly dominated by *Pseudomonas*, *Klebsiella*, *Cedecea*, *Azospirillum* and *Agromyces* compared to the midguts (Figure 5a). Similarly, the ovaries were differentially abundant in *Pseudomonas*, *Aeromonas*, *Agromyces* and *Azospirillum* compared to the midguts (Figure 5b). When comparing the relative abundances of bacteria genera between ovaries and salivary glands, the ovaries harbored more *Cutibacterium*, *Acinetobacter*, *Chryseobacterium*, *Aeromonas*, *Flectobacillus* and *Yersina,* while the salivary glands harbored more *Klebsiella* and *Cedecea* (Figure 5c).

**Figure 6:**
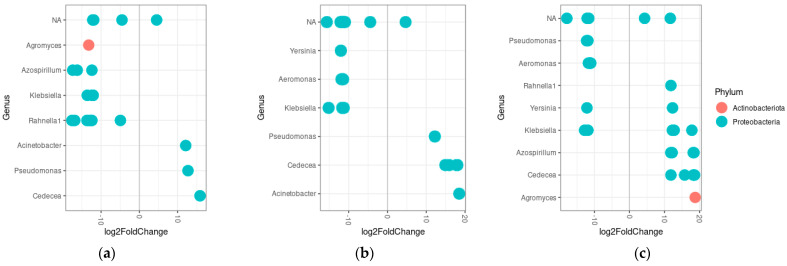
Differential abundance of genera in different strains of *An. funestus* across all three tissues. (**a**) Differential abundance between FANG (left) and FUMOZ (right), (**b**) differential abundance between FANG (left) and FUMOZ-R (right) and (**c**) differential abundance between FUMOZ (left) and FUMOZ-R (right). Numbers on either side of the 0 represent the log2fold change of bacterial genera that are significantly different at an alpha value of 0.01. Each dot represents a single OTU.

In terms of strain differences in the differential abundance of bacterial genera, FANG was more dominated by *Agromyces*, *Azospirillum*, *Klebsiella* and *Rahnella* compared to FUMOZ and *Yersinia*, *Aeromonas* and *Klebsiella* when compared to FUMOZ-R (Figure 6a,b). Both FUMOZ and FUMOZ-R were differentially abundant in *Acinetobacter*, *Pseudomonas* and *Cedecea* when compared to FANG (Figure 6a,b). When comparing FUMOZ and FUMOZ-R, FUMOZ was differentially abundant in *Pseudomonas* and *Aeromonas,* while FUMOZ-R was differentially abundant in *Rahnella*, *Azospirillum*, *Cedecea* and *Agromyces* (Figure 6c).

## 4. Discussion

The alpha diversity (species richness and evenness) was significantly higher for salivary glands as compared to the midguts and ovaries. More diverse and uniquely associated bacteria in the salivary glands compared to the midguts of *Anopheles* mosquitoes have been reported before [31,41]. For example, salivary glands of *An. culicifacies* habored the phyla Armatimonadetes, Cyanobacteria/Chloropyta, Chlorobi, Chlorofelxi, Planctomycetes, Nitrospira and Fibrobacter, which were not found in the midguts [31]. When looking at beta diversity, the clustering is statistically different based both on the tissues and the strains. Furthermore, since the salivary glands and the midguts of mosquitoes both form part of the mosquito digestive system, it is expected that the midguts and salivary glands share more bacterial genera (13) and species (27) than midguts and ovaries. Sharma et al. [31] reported 36 genera being shared between the salivary glands and midguts of *An. culicifacies*. The shared bacteria and clustering were further highlighted in the differential abundances between tissues, as well as between the strains.

Mosquito species and strain differences in alpha and beta diversity have been found to be more dependent on environmental differences, such as different breeding sites, rather than inherent species or strain differences [42,43,44]. For example, laboratory strains and field strains of *An. gambiae* exhibited different microbial compositions, where Proteobacteria represented more than 90% of the bacterial gut content in the mosquitoes from the wild, whereas Flavobacteriia (*Elizabethkingia* spp.) dominated 95% of laboratory-reared *An. gambiae* [43]. However, in the present study, despite being reared similarly in the same facility, the alpha diversity of FANG was statistically different to that of FUMOZ and FUMOZ-R, with FANG exhibiting the lowest alpha diversity when compared to FUMOZ and FUMOZ-R. The difference in diversity could be related to insecticide resistance. Microbiota differences have been reported between DDT-susceptible and -resistant *An. arabiensis*, between fenitrothion- and pyrethroids alphacypermethrin-susceptible and -resistant *Anopheles albimanus*, and between permethrin-susceptible and -resistant *An. gambiae* [27,28,29,45]. Interestingly, insecticide resistance has generally been associated with a decrease in the diversity of microbiota, while the opposite was observed in the present study [27,28,29,45]. The difference in alpha and beta diversity is therefore likely also influenced by the origins of the strains. FANG originated from Southern Angola, rather than Southern Mozambique, where FUMOZ and FUMOZ-R originated. Thus, FANG may harbor genetic differences relating to the core functions of the strain, which alter its core midgut microbiota. It is worth noting that despite the lower alpha diversity, FANG did have more differentially abundant bacterial species than both FUMOZ and FUMOZ-R.

However, regardless of the differences in richness, evenness, overlaps and abundance of microbial communities between different *An. funestus* strains and tissues, the top five dominating genera across all tissues and strains were *Elizabethkingia*, *Acinetobacter*, *Aeromonas*, *Cedecea* and *Yersinia*. Adult *An. coluzzii* and *An. gambiae* have been found to be dominated (~90%) by the phylum Proteobacteria [46]. Similar findings were observed in the present study, where four of the five most dominating genera across all three tissues belonged to the phylum Proteobacteria. Thus, *Elizabethkingia*, *Acinetobacter*, *Aeromonas*, *Cedecea* and *Yersinia* species likely form part of the core microbiota in *An. funestus*.

Infective mosquitoes are females that have taken an infected blood meal and have lived long enough for the *Plasmodium* parasite to have completed its life cycle and developed to the sporozoite stage in the salivary glands. This temperature-dependent development cycle means that only older females are of epidemiological significance, as young females will not be able to transmit the parasite [47,48]. Thus, due to the significance of older blood-fed female mosquitoes in the transmission of *Plasmodium*, the present study evaluated the microbiota found in older blood-fed females. This is likely why the dominating bacterial species in the present study was *Elizabethkingia anophelis*. The dominance of *Elizabethkingia* in the midgut has been found in *Anopheles gambiae* [43,49,50,51,52], *An. stephensi* [42,53] and *Aedes aegypti* [54]. Furthermore, *Elizabethkingia* has also been observed to be passed from mother to larva by attaching to the egg surface [12,51]. Thus, it is likely to be found beyond the midgut, as observed in the present study, where *Elizabethkingia* also dominated the ovaries and salivary glands across all three strains of *An. funestus*.

Older female *An. funestus* mosquitoes benefit from *E. anophelis* for several reasons. Firstly, *E. anophelis* possesses a hemolytic ability, which aids the mosquito in digestion after a blood meal [52]. Secondly, *E. anophelis* is capable of using the mevalonate (MVA) pathway instead of the 2Cmethyl-D-erythritol 4-phosphate (MEP) pathway that most eubacteria use to synthesize isoprenoids [52]. This ability of *E. anophelis* is advantageous because MEP pathways rely on the ability to form Fe–S complexes, which can be destroyed by nitric oxide [55,56]. Since the production of nitric oxide is essential to mosquito anti-parasitic and anti-bacterial responses, the ability of *E. anophelis* to use the MVA pathway instead of the MEP pathway ensures that the immune response of the host mosquito is not compromised by isoprenoid production [52].

The other dominating bacterium of note that was present across all tissues and all three mosquito strains was *Acinetobacter*. *Acinetobacter*, together with *Elizabethkingia*, and members of the Enterobacteriaceae family, also represented the most abundant taxa (80%) and the highest diversity in *Anopheles* mosquitoes 24 h after a blood meal [41]. Similarly, *Acinetobacter* (Proteobacteria) was the most dominant, being present in 53% of the abdomens of five *Anopheles* species from Vietnam, which included *Anopheles barbumbrosus*, *Anopheles crawfordi*, *Anopheles dirus*, *Anopheles maculatus* and *Anopheles gigas* [44]. Wild samples of the Funestus group from Vietnam, *Anopheles jeyporiensis*, *Anopheles minimus* and *An. Pampanai,* have also been shown to be dominated by Proteobacteria, Firmicutes and Actinobacteria [57]. Like *E. anophelis*, *Acinetobacter* has been linked to blood digestion and nectar assimilation in *Aedes albopictus* [14]. Moreover, the other relatively dominant bacteria in the present study, such as *Klebsiella*, *Serratia*, and *Pseudomonas*, have also been shown to be more capable of coping with oxidative stress after a blood meal [50]. Thus, the core microbiota of *An. funestus* is dependent on the core functions relating to its life parameters, such as blood digestion, rather than being due to the differences in strain and species.

## 5. Conclusions

The present study further expanded on current microbiota studies in important mosquito vectors by examining the core microbiota across tissues of vector-control importance in the major African vector, *An. funestus,* for the first time. Due to the crucial role that *Elizabethkingia* plays in older female mosquitoes, including blood digestion and fecundity, coupled with the fact that it can be transmitted vertically, a lot of attention has been paid to the use of *Elizabethkingia* as a paratransgenic tool [13,50,51,52,58]. The observation of the dominance of *Elizabethkingia* across all tissues and strains of *An. funestus* in the present study contributes towards a better understanding of potential paratransgenic tools for the control of *An. funestus* in Africa.

## Data Availability

The data presented in this study are available on request from the corresponding author.

## Supplementary Materials

The following supporting information can be downloaded at: https://www.mdpi.com/article/10.3390/tropicalmed9040084/s1, Table S1: Tissue overlap at the genus level; Table S2: Tissue overlap at the species level; Table S3: Strain overlap at the genus level; Table S4: Strain overlap at the species level; Table S5: Related abundance of all species; Table S6: Related abundance of top 50 taxa.
